# Methamphetamine Use in HIV-infected Individuals Affects T-cell Function and Viral Outcome during Suppressive Antiretroviral Therapy

**DOI:** 10.1038/srep13179

**Published:** 2015-08-24

**Authors:** Marta Massanella, Sara Gianella, Rachel Schrier, Jennifer M. Dan, Josué Pérez-Santiago, Michelli F. Oliveira, Douglas D. Richman, Susan J. Little, Constance A. Benson, Eric S. Daar, Michael P. Dube, Richard H. Haubrich, Davey M. Smith, Sheldon R. Morris

**Affiliations:** 1 University of California San Diego, La Jolla, CA, United States; 2 La Jolla Institute for Allergy & Immunology, La Jolla, CA, United States; 3 Veterans Affairs San Diego Healthcare System, La Jolla, CA, United States; 4 Los Angeles Biomedical Research Institute at Harbor-UCLA Medical Center, Torrance, CA United States; 5 University of Southern California Keck School of Medicine, Los Angeles, CA, United States

## Abstract

We investigated the associations between methamphetamine (meth) use, immune function, and the dynamics of HIV and cytomegalovirus [CMV] in the blood and genital tract of HIV-infected ART-suppressed subjects. Self-reported meth use was associated with increased CD4^+^ and CD8^+^ T-cell proliferation (Ki67^+^, *p* < 0.005), CD4^+^ T-cell activation (CD45RA^–^CD38^+^, *p* = 0.005) and exhaustion (PD-1^+^, *p* = 0.0004) in blood, compared to non-meth users. Meth use was also associated with a trend towards higher blood HIV DNA levels (*p* = 0.09) and more frequent shedding of CMV in seminal plasma (p = 0.002). To explore possible mechanisms, we compared *ex vivo* spontaneous and antigen-specific proliferation in PBMC collected from subjects with and without positive meth detection in urine (Utox+ vs. Utox-). Despite higher levels of spontaneous proliferation, lymphocytes from Utox+ meth users had a significantly lower proliferative capacity after stimulation with a number of pathogens (CMV, candida, mycobacterium, toxoplasma, HIV, *p* < 0.04 in all cases), compared to Utox- participants. Our findings suggest that meth users have greater proliferation and exhaustion of the immune system. Meth use is also associated with a loss of control of CMV replication, which could be related to loss of immune response to pathogens. Future studies should consider meth use as a potential modulator of T-cell responses.

Methamphetamine (meth) is a widely used recreational drug in North America^1^,^2^,^3^, and its use is highly prevalent among HIV-infected men who have sex with men (MSM)^4^. Meth can be self-administered intravenously, by nasal inhalation, smoking, anal insertion, or oral consumption in doses ranging from 250 to 500 mg for occasional users to as much as 1 gram by chronic addicts. Users also tend to consume meth in ‘binges’, and, as the drug has a half-life of 12 hours, this can lead to extremely high levels in blood^5^,^6^. Despite substantial evidence of the effects of meth use on the central nervous system and its association with neurocognitive impairment, the effects of meth on the immune system have not been extensively described. In murine models, meth has been described as an immunosuppressive agent by inhibiting antigen presentation, impairing phagocytosis and reducing the microbicidal capacity of macrophages^7^,^8^. Furthermore, meth exposure of primary human T cells *in vitro* induces in mitochondrial oxidative damage resulting in cellular dysfunction^9^.

In HIV-infected individuals, meth use is associated with delayed viral suppression after the initiation of antiretroviral therapy (ART), higher levels of blood HIV RNA, increased frequency of drug resistance mutations, and accelerated progression to AIDS^10^,^11^,^12^,^13^,^14^. Moreover, meth use is associated with significantly increased risk of other infectious diseases, HIV transmission, and mortality related to suicide and drug overdose^15^,^16^,^17^,^18^,^19^. It is unclear if the associations between meth use and HIV disease progression and transmission are purely a consequence of reduced ART adherence, poor nutrition, and increased risk behaviors associated with meth consumption^12^,^20^, or if there is a biological mechanism underlying these associations.

*In vitro*, meth is associated with increased cellular HIV transcription, immune activation and increased expression of the surface HIV co-receptor CCR5^21^,^22^,^23^,^24^ Since activated T-cells undergo extensive cell division and differentiation^25^, and the CCR5 co-receptor is an important co-factor in modulating cellular infection with HIV^26^, we hypothesized that all these mechanisms might enhance HIV infection and contribute to maintaining the HIV reservoir size. Despite these effects of meth *in vitro*, little is known about the outcome of meth *in vivo* on T lymphocytes and macrophages and how it may further compromise immune function in the setting of HIV infection.

Here, we investigated a cohort of 50 chronically HIV-infected MSM virologically suppressed on long-term ART who were well characterized in terms of: ART use, meth use, other drug use, and disease state to determine the relationships between meth use, levels of immune activation and proliferation, levels of CCR5 expression on T cells and macrophages, and the size and transcriptional activity of the viral HIV reservoir. We also evaluated the effect of meth on the function of T-cells by measuring *ex vivo* proliferation capacity of PBMCs from subjects with meth present in their urine (urine toxicology positive) compared to meth negative controls after stimulation with antigens from various pathogens.

## Results

### *In vivo* results from the California Collaborative Treatment Group (CCTG) samples

#### Baseline characteristics CCTG Cohort participant

Of 50 HIV-infected MSM virologically suppressed on ART included in this study, 16 individuals reported regular meth use over the 12 months of follow-up. Among meth group, meth use was reported in 40% [IQR:21–79%] of all evaluated monthly surveys. Eleven individuals reported consumption of meth in the month immediately preceding sample collection. Compared to non-users, meth users more frequently reported the use of other recreational drugs such as marijuana, cocaine and other club drugs (*p* < 0.02 in all cases). Group characteristics are summarized in Table 1. The demographic and clinical parameters were mostly balanced between both groups; however, meth users were more commonly on regimens containing PI than the control group (*p* = 0.02). The majority (88%) of participants self-reported levels of ART adherence >90% during the preceding month with no difference between meth groups. The proportion of meth users with cytomegalovirus (CMV) shedding in seminal plasma was higher than in the non-meth user group (*p* = 0.002, Table 1). Although only 4 participants had HIV shedding in the genital tract in this cohort, there was a trend towards a higher frequency of genital HIV shedding in meth users (*p* = 0.09, Table 1). No difference was observed for Epstein-Barr virus (EBV) shedding. No differences were found in any sexually transmitted infections (STI) (Including Gonorrhea, Chlamydia, Syphilis, trichomonas, mycoplasma) between groups.

#### Analysis of T-cell maturation subsets

Due to the increased proportion of genital tract CMV-shedders in the meth use group and the described association between CMV-shedding and the enrichment of transitional memory (T_tm_) and effector memory (T_em_) in CD4^+^ T-cell compartment^27^, we analyzed the frequencies and absolute counts of CD8^+^ and CD4^+^ T-cell subsets (naive [T_n_], central memory [T_cm_], T_tm_ and T_em_). There was no difference in the distribution (frequencies and absolute counts) of CD8^+^ and CD4^+^ T-cell subsets between meth and non-meth users (Supplementary Fig. 1). There was, however, a weak trend towards increased frequencies and absolute counts of circulating T_tm_ subset in CD4^+^ T cells in the meth user group but this did not reach statistical significance (*p* < 0.1 in both cases).

#### Analysis of T-cell activation

**Figure 1:**
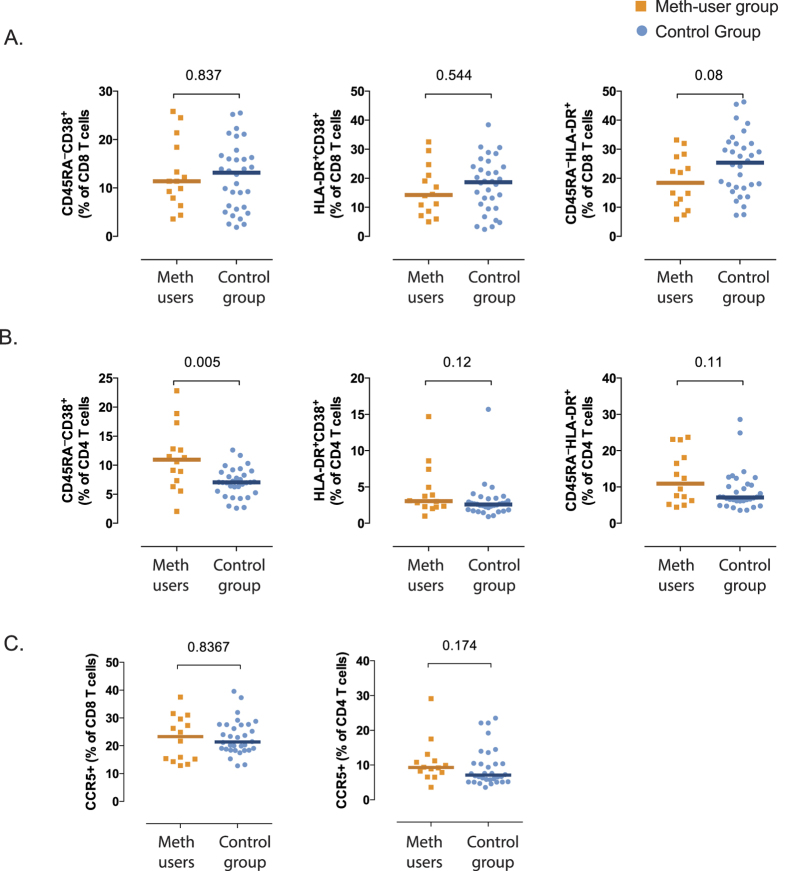
Effect of meth-use on immune activation and CCR5 expression on HIV ART-suppressed individuals. Immune activation markers and CCR5 expression in CD4 and CD8 T cells were assessed by flow cytometry using frozen PBMCs from the CCTG592 cohort. Comparison of CD8^+^ (**Panel A**) and CD4^+^ (**Panel B**) activation markers (measured as frequencies of CD45RA^–^CD38^+^, HLA-DR^+^CD38^+^ and CD45RA^–^HLA-DR^+^) and frequencies of CCR5 expression in total CD8^+^ and CD4^+^ T cells (**Panel C**) are plotted for meth-users (orange squares, n = 16) and control group (blue circles, n = 34). Individual and median values are shown. Two-sided *p*-values (Mann Whitney U test) are indicated.

Increased activation of CD4^+^ and CD8^+^ T cells, especially CD38 expression, is a hallmark of HIV infection that is associated with both progression to AIDS and response to ART in treated individuals^28^,^29^,^30^ 1. As naïve T cells express CD38 without activation, we analyzed activation reflected by co-expression of CD38 and HLA-DR or the expression of these markers singularly in memory lymphocytes (CD45RA^–^). There were no differences in CD8^+^ T-cell immune activation between groups, as measured by the frequency of CD45RA^–^CD38^+^ or HLA-DR^+^CD38^+^ (Fig. 1A), but there was a trend towards higher levels of CD45RA^–^HLA-DR^+^ in the non meth users compared to the users (*p* = 0.08, Fig. 1A). In contrast, immune activation in CD4^+^ T cells was significantly higher in the meth user group compared to non-users, as measured by the frequency of CD45RA^–^CD38^+^ (*p* = 0.005, Fig. 1B). While CD4^+^ T cells of meth users expressed higher levels of HLA-DR^+^CD38^+^ and CD45RA^–^HLA-DR^+^, this difference was not significant (*p* = 0.12 and *p* = 0.11 respectively, Fig. 1B).

Since genital CMV-shedding has been associated with increased immune activation^31^, we stratified both meth and control groups by seminal CMV-shedding status. In this sub-analysis, we observed that CD45RA^–^CD38^+^ in CD4 T cells was significantly increased in both meth user groups (i.e. meth+/non-CMV-shedders and meth+/CMV-shedders) compared to controls (Supplementary Fig. 2). However, this analysis was limited by the small sample size and deserves further investigation using a larger cohort of individuals.

Since treatment of macrophages with meth *in vitro* reportedly induces up-regulation of CCR*5* expression and increases frequency of infection with HIV^21^, we explored the *in vivo* effect of meth-use on this marker on T-cells. Despite the observed *in vitro* effects, we did not observe any difference in CCR5 expression between meth use groups in either CD4^+^ or CD8^+^ T cells (Fig. 1C). We also evaluated the percentage and mean fluorescence intensity (MFI) of CCR5 expression on monocytes, and similar to T cells, we did not find any differences between groups in the expression of CCR5 on monocytes (Table 2). The levels of sCD14 and sCD163, soluble markers of monocyte activation, also were not statistically different between groups (Table 2). Overall, these results suggest that a history of meth use is not associated with a sustained level of monocyte activation.

#### Analysis of T-cell proliferation

**Figure 2:**
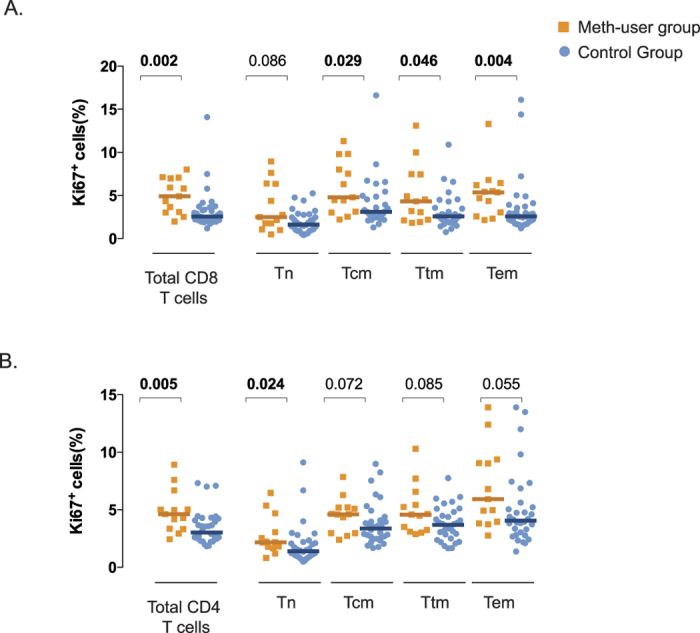
Effect of meth-use on T-cell proliferation in HIV-infected ART-suppressed individuals. T-cell proliferation (Ki67^+^) was assessed by flow cytometry using frozen PBMCs from the CCTG592 cohort. Proliferation of CD8^+^ (**Panel A**) and CD4^+^ (**Panel B**) of total or T-cell subsets (Tn, Tcm, Ttm and Tem) from meth-users (orange squares, n = 16) and control group (blue circles, n = 34) are plotted. T-cell subsets in CD8 and CD4+ T cells were defined as: Tn, CD45RA^+^CD27^+^CCR7^+^; Tcm, CD45RA^–^CD27^+^CCR7^+^; Ttm, CD45RA^–^CD27^+^CCR7^–^; Tem, CD45RA^+/–^CD27^–^CCR7^–^. Individual and median values are shown. Two-sided *p*-values (Mann Whitney U test) are indicated.

To investigate the levels of ongoing proliferation in total and T lymphocyte subsets we quantified the expression of Ki67, a nuclear antigen found in cycling cells. Total CD8^+^ T cells of reported meth users showed significantly higher percentages of Ki67 expression compared to non-users (*p* = 0.002, Fig. 2A). We also examined Ki67 expression among the different CD8^+^ T-cell maturation subsets. The proportion of proliferating CD8^+^ T_cm_, T_tm_ and T_em_ cells were significantly higher in meth users compared to non-users (*p* = 0.03, *p* = 0.05 and *p* = 0.004, respectively, Fig. 2A) and the proportion of cycling naïve CD8^+^ T cells tended to be higher in the meth group (*p* = 0.09). Similarly, total CD4^+^ T cells showed higher frequencies of Ki67^+^ cells in meth user group (*p* = 0.005, Fig. 2B). Among subsets, the meth users presented significantly higher levels of proliferating naïve CD4^+^ T-cells (*p* = 0.02), while the other subsets (T_cm_, T_tm_ and T_em_) tended to have higher frequencies of Ki67^+^, but these differences did not reach statistical significance (*p* = 0.07, *p* = 0.08 and *p* = 0.06, respectively, Fig. 2B).

Since the presence of genital tract CMV shedding has been associated with higher levels of CD4^+^ and CD8^+^ T-cell proliferation^27^ and meth users had a higher frequency of CMV-shedding than non-meth users, we performed a multivariate analysis adjusting for CMV shedding. In this analysis, meth use remained independently associated with CD4^+^ and CD8^+^ T-cell proliferation (*p* < 0.05 in both cases).

#### Immune exhaustion

**Figure 3:**
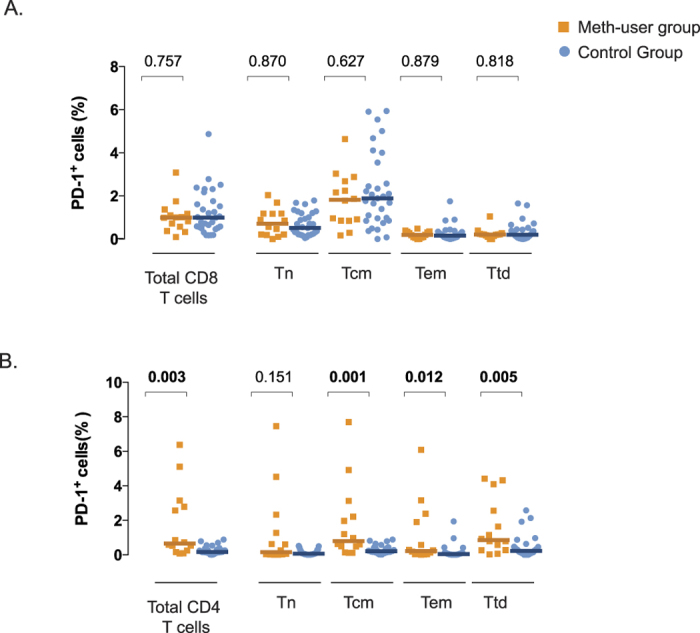
Effect of meth-use on T-cell proliferation in HIV-infected ART-suppressed individuals. T-cell exhaustion (PD-1^+^) was assessed by flow cytometry using frozen PBMCs from the CCTG592 cohort. Exhaustion levels of CD8^+^ (**Panel A**) and CD4^+^ (**Panel B**) of total or T-cell subsets (Tn, Tcm, Tem and Ttd) are plotted from meth-users (orange squares, n = 16) and control group (blue circles, n = 34). T-cell subsets in CD8^+^ and CD4^+^ T cells were defined as: Tn, CD45RA^+^CD27^+^CD28^+^; Tcm, CD45RA^–^CD27^+^CD28^+^; Tem, CD45RA^+/–^CD27^+^CD28^–^ and CD45RA^+/–^CD27^–^CD28^+^ for CD8^+^ and CD4^+^, respectively; Ttd, CD45RA^+/–^CD27^–^CD28^–^. Individual and median values are shown. Two-sided *p*-values (Mann Whitney U test) are indicated.

As persistent lymphocyte proliferation can lead to cellular exhaustion, we examined the levels of PD-1, considered a marker of lymphocyte exhaustion. We did not find any difference between meth users and non-users in the frequency of PD-1^+^ total CD8^+^ T-cells or any of the CD8^+^ T-cell subsets (Fig. 3A). However, significantly higher frequencies of PD-1 expression were observed for total CD4^+^ T-cells from meth users (Fig. 3B, *p* = 0.003). When CD4^+^ T-cell subsets were analyzed, significantly higher levels of PD-1^+^ expression were detected in T_cm_, T_em_ and T_td_ among meth users (Fig. 3B, *p* < 0.002 in all cases). In a multivariate analysis, levels of CD4^+^ T-cell exhaustion remained independently associated with meth use after adjusting for the presence of CMV (*p* = 0.004).

#### Analysis of HIV DNA

**Figure 4:**
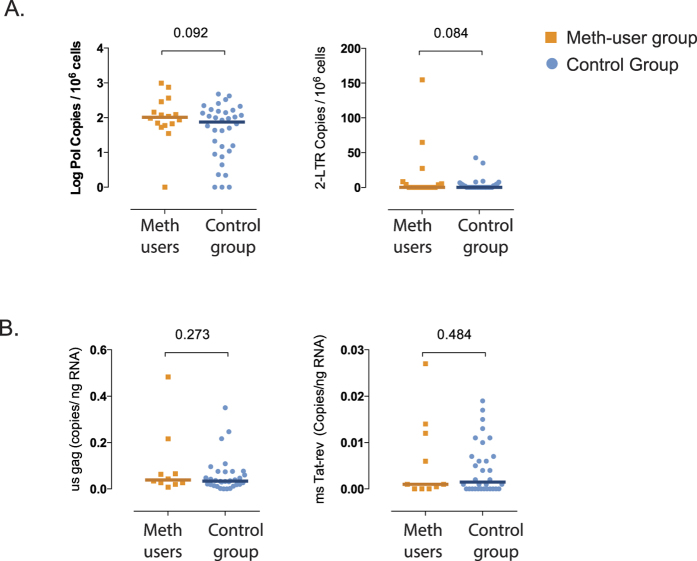
Characterization of HIV cellular reservoir in meth users and control group. Total DNA and RNA were extracted from 5 × 10^6^ frozen PBMCs and HIV DNA and RNA levels were quantified by ddPCR. For meth-users (orange squares, n = 16) and control group (blue circles, n = 34), levels of total HIV DNA and 2-LTRs were normalized per million CD4^+^ T cells (**Panel A**) and levels of unspliced (gag) and multiply spliced (tat-rev) HIV RNA were normalized per ng of RNA (**Panel B**). Individual and median values are shown. Two-sided *p*-values (Mann Whitney U test) are indicated.

Increased inflammation and CD4^+^ T-cell proliferation have been associated with maintenance of the HIV cellular reservoir during suppressive ART^32^. Therefore, we investigated the effect of meth use on the levels of HIV DNA and cell-associated HIV RNA. A trend towards higher levels of total HIV DNA was found in the meth user group (*p* = 0.09, Fig. 4A), as well as a trend for increased levels of 2-LTR circles (*p* = 0.08, Fig. 4A). No significant difference was observed for cell-associated HIV RNA (unspliced [gag] and multiply spiced [tat/rev], Fig. 4B) or transcriptional activity (estimated as the cellular HIV RNA/HIV DNA ratio, data not shown) between meth user and non-user groups. Since genital tract CMV-shedding has also been associated with higher total HIV DNA levels^31^, we performed a multivariate analysis to adjust for this parameter. After this adjustment for CMV shedding, the increased levels of HIV DNA observed among meth users was no longer significant (*p* = 0.38), suggesting that CMV shedding has a stronger effect on HIV DNA levels than a history of meth use.

#### Protease inhibitors

As noted above, almost 90% of meth users were on a PI-based regimen (compared to 50% of non meth users, Table 1). Some studies have suggested that PI-treated individuals display higher levels of inflammation, immune activation^33^,^34^ and residual HIV replication^35^,^36^. We therefore investigated if PI use was also associated with any of the parameters significantly associated with meth use, stratifying subjects based on the PI containing ART regiment (PI and non-PI use groups, n = 30 and n = 20, respectively). PI-use was associated with higher CD8^+^ T-cell proliferation (*p* = 0.05), CD4^+^ T-cell exhaustion (*p* = 0.03) and proviral HIV reservoir (*p* = 0.03). In a multivariate analysis including meth use and PI use, meth use remained independently associated with CD8^+^ T-cell proliferation and CD4^+^ T-cell exhaustion (*p* < 0.05 in both cases), but not with proviral HIV DNA levels (*p* = 0.26).

#### Effects on recent meth-use

We identified 11 individuals out of 16 who self-reported meth use during the month immediately preceding sample collection. We performed a sensitivity analysis on this subset of individuals (n = 11) compared with non-meth users (n = 34). We were able to confirm our previous findings: increased levels of CD4^+^ (in total and T_n_ subset) and CD8^+^ T–cell proliferation (total and all subsets), increased CD4^+^ T-cell activation (measured as the frequency of CD45RA^–^CD38^+^) and exhaustion (Supplementary Table 1). No differences were observed between groups on the levels of HIV DNA. However, these results were limited by the lower number of subjects with recent meth use.

### *Ex vivo* results from the HIV Neurobehavioral Research Program (HNRP) samples

#### Acute effects of meth on lymphocyte function

**Figure 5:**
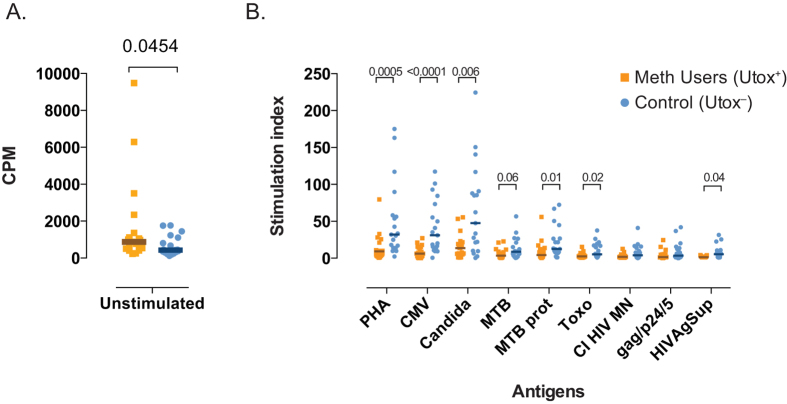
*Ex vivo* T-cell proliferative responses to antigen stimuli. Fresh PBMCs from HIV infected individuals from meth users (urine toxicology positive, orange squares, n = 19) and non-meth users (urine toxicology negative controls, blue squares, n = 18) were cultured in triplicates for 7 days in absence (**Panel A**) or in presence of phytohemagglutinin (PHA) and different antigen: cytomegalovirus (CMV), Candida, *Mycobacterium tuberculosis* (MTB), MTB protein, Toxoplasma (Toxo), HIV gag/p24/p5 and heat-inactivated (1 hour, 56 °C) supernatant of HIV infected T cells (HIVAgSup) (**Panel B**). Cells were pulsed with [3H]-thymidine 24 hours prior to proliferation analysis. For unstimulated wells in panel A, the uptake of [3H]-thymidine is presented as counts per minute (cpm) and values are the average of 3 wells. For panel B, stimulation index (SI) was calculated as a ratio of the mean cpm as measured for each stimulus (PHA and other antigens) to the mean cpm of unstimulated control. Individual and median values are shown. Two-sided *p*-values (Mann Whitney U test) are indicated.

Memory T-cell responses to mitogen (PHA) and opportunistic pathogen antigens were evaluated *ex vivo* using PBMC from HIV infected individuals (n = 19) with detectable meth in their urine (UTox^+^) at a scheduled clinic visit at the HNRP. Among these individuals, the median CD4^+^ T cell count was 438 [283–658] cells/μl and median log_10_ HIV RNA was 3.7 [3.1–4.5] copies/mL. We included a control group of HIV-infected individuals from the same cohort who did not use meth (UTox^–^, n = 18) but who were matched for HIV RNA levels (median log_10_ HIV RNA 3.0 IQR: 2.3–3.8 copies/mL) and CD4^+^ T-cell counts (median 402 IQR:271–618 cells/μl). Constitutive proliferation of T cells was significantly higher in UTox^+^ meth users than in UTox^–^ participants (*p* = 0.045, Fig. 5A). However, UTox^+^ meth users demonstrated significantly reduced proliferative responses (i.e. lower stimulation index) after stimulation with PHA (*p* = 0.0005), CMV (*p* < 0.0001), Candida (*p* = 0.006), MTB protein (*p* = 0.01), Toxoplasma (*p* = 0.02) and supernatant of HIV infected cells (HIVAgSup) (*p* = 0.04, Fig. 5B) compared to UTox^–^ subjects. Only a trend to lower proliferative response was observed for MTB (*p* = 0.06), and there was no significant difference between groups when the PBMCs were stimulated with chemically inactivated HIV MN and purified gag/p24/5.

## Discussion

Previous studies of meth use by HIV-infected individuals have suggested that meth use is associated with faster disease progression, neurocognitive impairment, inflammation, and profound suppression of immune function^12^,^37^. Since meth use is also linked to reduced adherence to ART and a failure to suppress HIV replication, it has been difficult to differentiate between direct effects of meth and those resulting from residual HIV replication related to non-adherence^12^.

For the *in vivo* study, we included HIV-infected participants from the CCTG cohort who had HIV RNA levels suppressed in blood during long-term ART with the goal of isolating the effect of meth use on immune activation, T-cell proliferation and the HIV cellular reservoir. We found that these well-characterized meth users had significantly increased levels of CD4^+^ and CD8^+^ T-cell proliferation and increased activation (especially measured as the frequency CD45RA^–^CD38^+^) and exhaustion of CD4^+^ T-cells. To further concentrate on the effects of meth use, we performed a sensitivity analysis on a subset of 11 individuals from the CCTG cohort who self-reported meth use during the month immediately preceding the collection of blood and semen. In this small sub-group, we also found increased levels of T-cell proliferation, activation and exhaustion. Taken together, these results suggest that meth use may have relatively long-term (>30 days) consequences with respect to immune function, but longitudinal studies will be necessary to confirm these findings.

Two non-mutually exclusive mechanisms could drive the increased frequencies of T-cell proliferation, activation and exhaustion observed *in vivo*: 1) a direct effect of meth on T cells and, 2) CMV-associated immune activation^27^, since meth users shed CMV in the genital tract more frequently than non-meth users. As we previously reported^27^, CMV-shedding increases the frequency of Ki67 cells in CD4^+^ T-cells, and to some extent, in CD8^+^ T-cell compartment. In this study, we found a general and non-specific increase of proliferation in all T-cell subsets, which suggests a direct effect of meth in these cells *in vivo*. However, we cannot rule out an additional effect of CMV-shedding on T-cell proliferation levels, especially in CD4^+^ T cells^27^. Regarding immune activation, our results suggest that meth use and CMV-shedding increase the levels of activation markers independently and by different mechanisms. While CMV shedding is associated with increased levels of HLA-DR^+^CD38^+^ in CD4^+^ T cells overall, meth use is linked to an increase in the frequency of memory CD45RA^–^CD38^+^ CD4^+^ T cells. Therefore, both mechanisms might contribute and perhaps add to activation of the immune system, leading to an increased risk of morbidity and mortality^29^,^38^,^39^,^40^. Independent of the CMV-effects, more *ex vivo* studies will be required to determine the specific effects of meth on T-cell proliferation and activation.

Most of the meth users in our study were on PI-containing regimen, and higher levels of inflammation and immune activation are generally observed in subjects on PI-based regimens^33^,^34^. Although PI use has been associated with higher levels of CD8^+^ T-cell proliferation and CD4^+^ T-cell exhaustion, meth use still significantly contributed to the model after adjusting for PI use. It is possible that because meth-users have higher rates of Hepatitis C virus (HCV) co-infection, poor adherence to ART, and residual HIV load despite treatment, physicians may prefer PI-containing regimens, which have once daily dosing, high potency and a relatively high genetic barrier to resistance^41^.

Despite long term ART and fully suppressed HIV RNA in blood plasma, meth users tended to have higher levels of proviral HIV DNA in blood CD4^+^ cells and more frequent detection of HIV RNA in seminal plasma. However, the association of meth and higher proviral HIV DNA disappeared after adjusting for the presence of CMV shedding and PI use, suggesting that factors other than meth might play a larger role in the size and persistence of the HIV reservoir^27^. Alternatively, the meth-induced increased proliferation of CD4^+^ T cells could also contribute to an increased HIV DNA reservoir, due to a homeostatic proliferation of cells containing integrated HIV DNA^32^. Altogether, these findings could contribute to increased HIV transmission and worse HIV disease outcomes in subjects reporting meth use.

Further, to explore possible meth-related mechanisms of immune dysfunction, we performed *ex vivo* experiments using samples collected from UTox^+^ and UTox^−^ HNRP participants. Our data showed that, despite the increase of spontaneous T-cell proliferation in cells collected from UTox^+^ meth users, there was a clear concomitant loss of T-cell proliferative responses against various pathogens (i.e. CMV, HIV, Candida, MTB and Toxoplasma). This finding supports a hypothesis that meth use leads to poorer cytotoxic T lymphocyte (CTL)-mediated control of viral infections, like CMV and HIV, and may explain the higher rate of CMV and HIV shedding observed among meth users in the CCTG cohort. This observation was also corroborated in some animal studies, which have suggested that meth suppresses both innate and adaptive immunity^42^,^43^. For example, a similar effect of meth on lymphocyte function has been demonstrated in a mouse model that found the administration of meth accelerated the progression to death in mice infected with *Histoplasma capsulatum*, and this progression correlated with a decrease in the histoplasma-specific proliferative response^8^.

Several mechanisms could be involved in the reduction of specific proliferative responses. For instance, the treatment of murine cells with high doses of meth *in vitro* exerted direct immunosuppressive effect on dendritic cells and macrophages^7^. In these antigen presenting cells, meth inhibited antigen processing, presentation, and phagocytosis by collapsing the pH gradient across acidic organelles^7^. Another interesting mechanistic pathway of immune dysfunction may be oxidative damage to mitochondria by meth, which has been demonstrated *in vitro*, leading to impairment of lymphocyte function^9^. Interestingly antioxidants could attenuate the mitochondrial damage in this model^9^, suggesting that increased levels of reactive species from meth could be the cause of the diminished proliferative capacity of lymphocytes. Further studies need to be performed in humans to characterize specific meth-related deficits in lymphocyte and monocyte/macrophage functions.

**Figure 6:**
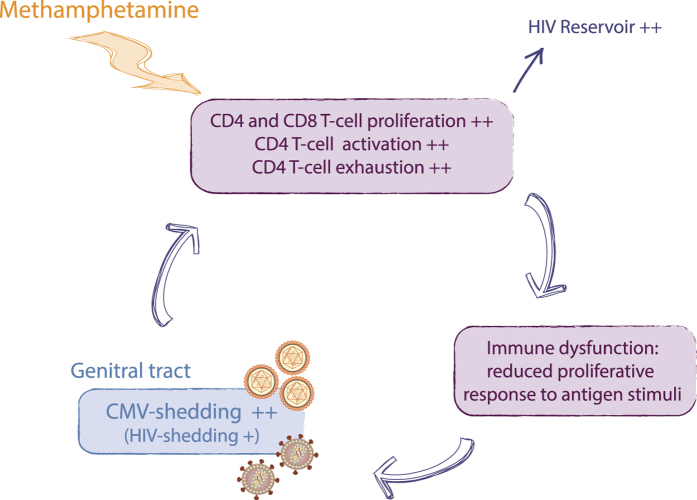
A model for the effect of meth use in HIV-infected ART-Suppressed individuals. Meth-use increases CD4^+^ and CD8^+^ T-cell proliferation as well as CD4 T-cell activation (CD45RA^–^CD38^+^) and exhaustion (PD-1^+^). This sustained effect may cause an immune dysfunction, such as a reduction of proliferative responses to antigen stimuli. The consequence is a loss of control of latent virus replication, such as CMV and HIV replication, which in turn will contribute to the increase immune activation, proliferation and exhaustion. The continuous proliferation/activation of CD4 T-cells as well as CMV-shedding could contribute to the increased HIV reservoir observed in meth users.

In summary, these studies demonstrate that the interplay between meth, CMV and HIV is complex. In particular, meth use in HIV-infected individuals may contribute to activation and exhaustion of the immune system, especially the CD4^+^ T-cell compartment, even when these individuals have HIV replication suppressed in blood plasma. Such ongoing proliferation exhausts immune cells, potentially preventing antigen specific T-cell proliferation and reducing cytotoxic T lymphocyte activity and antibody production. The consequence result could be failure to control reactivating endogenous pathogens, such as CMV (which is consistent with the increased shedding of CMV in the genital tract observed in the present study), and this persistent viral replication might further affect and exhaust the immune system, which in turn could diminish capacity to control virus replication (Fig. 6). The long-term clinical impact of this circular interaction likely explains in part why meth users have worse HIV disease outcomes despite use of effective ART.

## Material and Methods

### Ethics Statement

Ethical approval for this study (ClinicalTrials.gov Identifier: NCT01198418, grant title: *A Web-based Intervention Study to Reduce High-risk Sexual Behavior by Persons Living With HIV AIDS [PLWH]*) was obtained from the Offices of Human Research Protections Program of: the University of California San Diego, the Los Angeles Biomedical Research Institute at Harbor-UCLA Medical Center, and the University of Southern California in compliance with the Declaration of Helsinki. All determinations were performed with fully informed written consent from all participants involved in this study. All experiments were performed in accordance with approved guidelines and regulations.

### Participants, samples and clinical laboratory tests

Two distinct clinical cohorts were included in the described analysis for the *in vivo* (California Collaborative Treatment Group [CCTG] cohort) and *ex vivo* (HIV Neurobehavioral Research Program [HNRP] cohort) experimental part.

#### CCTG Cohort

The CCTG-592 trial is a prospective study of an internet-based behavioral intervention among MSM with ongoing transmission risk behaviors to reduce sexually transmitted infections. This study included baseline collection of paired blood and semen samples in a total of 179 HIV-infected MSM who were on or off ART^44^. For this cross-sectional study, we included baseline samples from a subset of 50 subjects selected sequentially from among those virologically suppressed on ART (plasma HIV RNA levels <50 copies/ml) on their last clinical visit (within 3 months prior to the collection of seminal and blood samples), and who had viable frozen PBMC aliquots available. Use of ART, meth and other drugs (marijuana, cocaine, heroin, alcohol and other “club” drugs, including Ecstasy, γ-hydroxybutyric acid [GHB], amyl nitrates, ketamine) were assessed monthly with a self-administered internet-based survey along with other risk behaviors for the 12 months of study follow-up. Since meth is highly addictive and recurrent use is common, meth use (or other drug-use) was defined as any reported consumption on at least one survey over the period of the study. All included participants were cytomegalovirus (CMV) seropositive^45^. Semen was collected and processed as previously described^46^,^47^. Blood absolute CD4^+^ T-lymphocyte counts were measured by flow cytometry, and HIV RNA levels in blood plasma were quantified by the Amplicor HIV Monitor Test (limit of detection 50 copies/mL, Roche Molecular Systems Inc).

#### HNRP cohort

*Ex-vivo* T-cell proliferation results were derived from 37 HIV-infected subjects enrolled in studies at the HRNP. Fresh peripheral blood mononuclear cells (PBMCs) were harvested for *ex vivo* antigen stimulation experiments from 19 HIV-infected individuals who tested positive for meth and also tested negative for cocaine, opiates, barbiturates, benzodiazepines, PCP, THC, and ETOH with urine toxicology. These individuals had HIV RNA levels ranging from <50 to 230,400 copies/mL and a median CD4^+^ T-cell count of 402 [271–618] cells/μl. All admitted use of meth within the past 36 hours. Eighteen HIV-infected control subjects from the same cohort who tested negative for all listed substances by urine toxicology and had no history of substance abuse were matched for HIV RNA levels and CD4^+^ T-cell counts.

### *In vivo* experiments performed on CCTG samples

#### Multiparameter Flow Cytometry Analysis

Immune activation, proliferation and exhaustion were assessed using frozen PBMCs from the CCTG592 cohort. Aliquots of 5 million PBMCs were quickly thawed at 37 °C, resuspended in RPMI supplemented with 20% FBS, incubated at room temperature for 20 minutes to allow cell recovery before staining. Cell viability was assessed using the LIVE/DEAD® Fixable Aqua Dead Cell Stain Kit (Life Technologies). Four subjects were excluded because their samples demonstrated <85% cell viability. Approximately 300,000–500,000 PBMC per tube were stained for cell surface markers prior to fixation, and when required, followed with permeabilization for intracellular protein detection (FOXP3/transcription factor Staining Buffer set, eBioscience). The stained cells were acquired on a FACS Canto (BD Biosciences). We used the following antibody combinations: (tube 1) HLA-DR–FITC (Clone G46-6), CD45RA–PE (Clone HI100), CD4–PerCP-Cy5.5 (Clone SK3), CD38–PE-Cy7 (Clone HB7), CD27–APC (Clone L128), CD3–APC-Cy7 (Clone SK7), CD8–V450 (Clone RPA-T8); (tube 2) Ki67–FITC (Clone B56), CD45RA–PE (Clone HI100), CD4–PerCP-Cy5.5 (Clone SK3), CCR7–PE-Cy7 (Clone 3D12), CD27–APC (Clone L128), CD3–APC-Cy7 (Clone SK7) and CD8–V450 (Clone RPA-T8); (tube 3) CCR5–FITC (Clone 2D7/CCR5), CD45RA–PE (Clone HI100), CD14–ECD (Clone RMO52), CD4–PerCP-Cy5.5 (Clone SK3), CCR7–PE–Cy7 (3D12), CD27–APC (Clone L128), CD3–APC-Cy7 (Clone SK3) and CD8–V450 (Clone RPA-T8); and (tube 4) CD57–FITC (Clone NK-1), CD45RA–PE (Clone HI100), CD28–ECD (Clone CD28.2), CD4–PerCP-Cy5.5 (Clone SK3), PD-1–PE-Cy7 (Clone EH12), CD27–APC (Clone L128), CD3–APC-Cy7 (Clone SK7), CD8–V450 (Clone RPA-T8). (All antibodies were from BD Biosciences, except CD14-ECD and CD28-ECD, which were from Beckman Coulter). The antibody combinations in Tube 1 were used to assess naïve (T_n_, CD45RA^+^CD27^+^), central memory (T_cm_, CD45RA^–^CD27^+^), effector memory (T_em_, CD45RA^–^CD27^–^) and terminally differentiated (T_td_, CD45RA^+^CD27^–^) CD4+ and CD8 T-cell subsets. With Tube 2 and 3, T-cell subsets were defined as: T_n_, CD45RA^+^CD27^+^CCR7^+^; T_cm_, CD45RA^–^CD27^+^CCR7^+^; T_tm_, CD45RA^–^CD27^+^CCR7^–^; and T_em_, CD45RA^+/–^CD27^–^CCR7^–^ in both the CD4^+^ and CD8^+^ subsets. T-cell subsets in tube 4 were defined as: T_n_, CD45RA^+^CD27^+^CD28^+^; T_cm_, CD45RA^–^CD27^+^CD28^+^; T_em_, CD45RA^+/–^CD27^+^CD28^–^ and CD45RA^+/–^CD27^–^CD28^+^ for CD8^+^ and CD4^+^, respectively; T_td_, CD45RA^+/–^CD27^–^CD28^–^. Immune activation was defined by CD38^+^HLA-DR^+^ or the expression of CD38 or HLA-DR in memory subsets. Proliferation and exhaustion was defined by expression of Ki67^+^ or PD-1^+^ respectively, in total CD4^+^ and CD8^+^ T-cell populations and subsets. Analyses of flow cytometry data were performed using Flow Jo software (version 9.6.2).

#### Soluble markers

Soluble CD14 (sCD14) and CD163 (sCD163) were quantified in plasma by ELISA (R&D systems and Thrillium, respectively), and interpreted as surrogate markers of monocyte activation.

#### Quantification of total and 2-Long Terminal Repeat (2-LTR) HIV DNA, and Herpesvirus DNA in PBMC and Semen

DNA was extracted from 5 million PBMC and 200 μl seminal plasma for each participant (QIAamp DNA Mini Kit, Qiagen, CA). For PBMC, total HIV DNA (pol) and 2-LTR circles were quantified by droplet digital PCR (ddPCR) from extracted DNA^48^, as described previously^27^. Copy numbers were calculated as the mean of replicate PCR measurements and normalized to one million CD4^+^ T cells as determined by RPP30 (total cell count) and flow cytometry (percentage of CD4^+^ T cells within total PBMCs). Levels of CMV and Epstein-Barr virus (EBV), were also measured by real-time PCR in the DNA extracted from seminal plasma, as described previously^46^.

#### Quantification of cellular HIV RNA in PBMC

Cellular HIV RNA (unspliced [gag] and multiply spliced [encoding for tat-rev]) was measured for the subset of 42 cohort participants with enough available PBMC to perform these analyses, as described previously^27^. Copy numbers were calculated as the mean of replicate PCR measurements, and normalized to total RNA as determined by A260/A280 absorptivity ratio using a NanoDrop 2000 spectrophotometer (Thermo Scientific).

### *Ex vivo* experiments performed on HNRP samples

#### *Ex vivo* T-cell proliferative responses to antigen stimuli

Fresh PBMCs from HIV-infected individuals were cultured with antigen or mitogen in triplicate in Iscoves media supplemented with 5% human AB serum for 7 days at 37 °C in 5% CO_2_ in presence of phytohemagglutinin (PHA) or pathogen antigens: heat-inactivated CMV AD-169 (grown by Dr. Rachel Schrier’s lab), Candida (Greer Biologics), *Mycobacterium tuberculosis* (MTB) protein (supplied by Dr. Henry Boom), Toxoplasma [Toxo] (donated by Dr. Sharon Reed), HIV gag/p24/p5 [Protein Sciences] and heat-inactivated (1 hour, 56 °C) supernatant of HIV infected T cells [HIVAgsupt], grown by Dr. Rachel Schrier’s lab). Chemically inactivated HIV MN was supplied by Dr. Jeffrey Lifson at NCI. PBMC cultured only with medium were used as unstimulated control. Cells were pulsed with [^3^H]-thymidine (0.5 ACi/well, NEN) 24 hours before harvest. Radioactivity was determined with a liquid scintillation counter (Packard Top Count). Data were presented as counts per minute (cpm) and triplicates were averaged. Stimulation index (SI) was calculated as the ratio of the mean cpm as measured for each stimulus (PHA and other antigens) to the mean cpm of unstimulated control.

### Statistics

Statistical analyses were performed using GraphPad Prism (version 6) and R statistical software. HIV RNA, CMV DNA, and EBV DNA levels were transformed to log_10_ values, and CMV genital shedding and presence of LTR were dichotomized (undetectable/detectable). Comparisons were made between the meth users and non-meth users using Fisher exact test (for dichotomous data) or Mann Whitney U test (for continuous data). To explore the cofounding effects in proliferation, immune exhaustion and HIV reservoir size, we adjusted our analysis for CMV-shedding in the genital tract and use of a protease inhibitor (PI) in the ART regimen using a multivariate fixed-effects regression model when one or both of these potential confounders had a p-value ≤ 0.1 in univariate analyses. Continuous variables were assessed for normalcy by the Shapiro-Wilk test and log-transformed if necessary.

## Additional Information

**How to cite this article**: Massanella, M. *et al*. Methamphetamine Use in HIV-infected Individuals Affects T-cell Function and Viral Outcome during Suppressive Antiretroviral Therapy. *Sci. Rep*. **5**, 13179; doi: 10.1038/srep13179 (2015).

## Supplementary Material

Supplementary Information

## Figures and Tables

**Table 1:** Participant characteristics.

	Meth^1^ n = 16	Control n = 34	*P*-value^5^
Age (years), median [IQR]	45 [36–49]	48 [40–54]	*0.11*
Race/Ethnicity, n(%)			
Caucasian	7(44)	14 (41)	*1.00*
Latino	4 (25)	13 (38)	*052*
African American	4 (25)	6 (18)	*0.72*
Other	1 (6)	1 (3)	*0.54*
Time on ART (years), median [IQR]	4.4 [2.2–5.9]	3.4 [1.7–5.7]	*0.51*
Nadir CD4^+^ T cell counts (cell/μl), median [IQR]	204 [120–300]	216 [60–386]	*0.94*
CD4^+^ T-cell counts (cell/μl), median [IQR]	577 [485–835]	652 [548–714]	*0.57*
CD4^+^%, median [IQR]	31 [25–36]	34 [28–37]	*0.56*
CD8^+^ T-cell counts (cell/μl), median [IQR]	852 [571–1113]	820 [507–939]	*0.27*
CD8%, median [IQR]	42 [38–52]	38 [31–46]	***0.08***
Ratio CD4/CD8, median [IQR]	0.72 [0.49–1.0]	0.83 [0.66–1.2]	***0.13***
Antiretroviral treatment, n(%)			
PI-based	**13 (87)**	**17 (50)**	***0.02***
NNRTI-based	4 (27)	16 (47)	*0.22*
Raltegravir containing regimen	1 (7)	7 (21)	*0.41*
Recreational drug use^1^, n (%)			
% of Positive surveys for Meth, median [IQR]	40 [21–79]	NA	
Marijuana^1^	11 (69)	9 (26)	***<0.01***
Cocaine^1^	8 (50)	3 (9)	***<0.01***
Other Club Drug 1,^ 2^	8 (50)	5 (15)	*0.01*
Alcohol1,^ 3^	5 (31)	7 (21)	*0.50*
Virus Shedding in semen, n (%)			
HIV	3 (19)	1 (3)	***0.09***
CMV	12 (75)	9 (26)	***<0.01***
EBV	4 (25)	8 (24)	*1.00*
Any other STI^4^, n (%)	2 (12.5)	3 (8.8)	*0.64*

^1^Drug-users reported consumption of the specified drug at least in one survey over the period of the study.

^2^Other club drugs: Ecstasy, GHB, Amyl nitrates, ketamine.

^3^5 or more alcoholic drinks on a single occasion >5 times/month.

^4^STI: sexually transmitted infections, including Gonorrhea, Chlamydia, Syphilis, Trichomonas, mycoplasma.

^5^Mann-Whitney U test.

**Table 2:** Monocyte activation.

	Meth^1^ n = 16	Control n = 34	*P*-value^2^
MFI^3^ of CCR5^+^CD14^+^, median [IQR]	2149 [1706–2912]	2294 [1730–2941]	*0.89*
sCD14, ng/mL, median [IQR]	800 [723.6–1056]	766.8 [586.9–888.9]	*0.21*
sCD163, ng/mL, median [IQR]	719.4 [516.2–1057]	724.0 [581.8–861.8]	*0.86*

^1^Drug-users reported consumption of the specified drug at least in one survey over the period of the study.

^2^Mann Whitney U test.

^3^MFI: mean fluorescence intensity.
