# Supplementary material for: Methamphetamine Use in HIV-infected Individuals Affects T-cell Function and Viral Outcome during Suppressive Antiretroviral Therapy
Source: Sci Rep. 2015 Aug 24;5:13179. doi: 10.1038/srep13179 (PMC4547398; doi:10.1038/srep13179)
Supplement: Supplementary Information [file srep13179-s1.pdf]

Methamphetamine Use in HIV-infected Individuals Affects T-cell Function and Viral Outcome during Suppressive Antiretroviral Therapy

Marta Massanella, Sara Gianella, Rachel Schrier, Jennifer Dan, Josué Pérez-Santiago, Michelli F. Oliveira, Douglas D Richman, Susan J Little, Constance A Benson, Eric S Daar, Michael P.Dube, Richard H Haubrich, Davey M Smith and Sheldon R. Morris

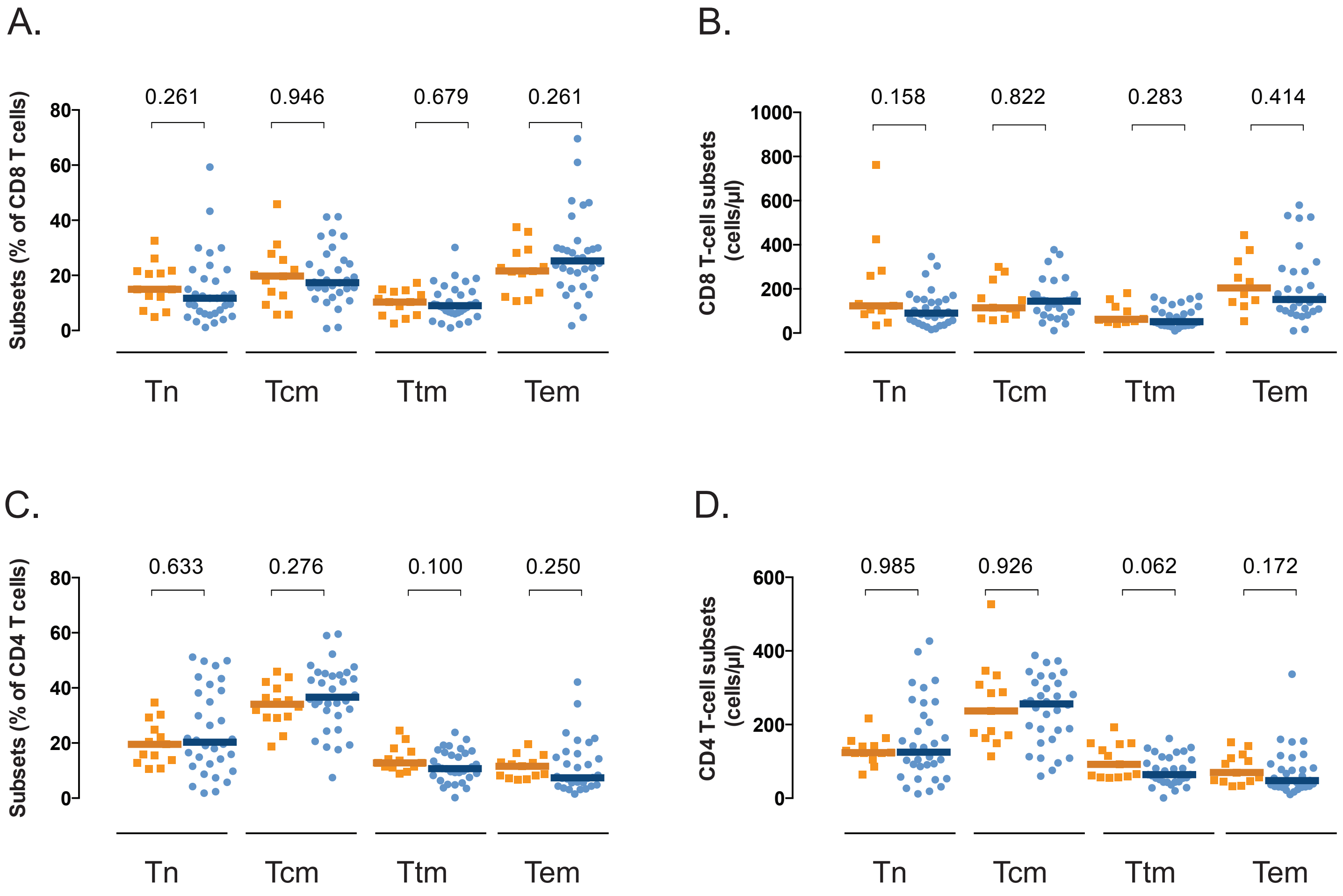

Supplementary Figure 1: Distribution of frequencies and absolut counts of CD8 (Panel A an B) and CD4 (Panel C and D) T-cell subsets in meth-users (orange squares) and control group (blue circles) are plotted. Individual and median values are shown. Two-sided p-values (Mann Whitney U test) are indicated.

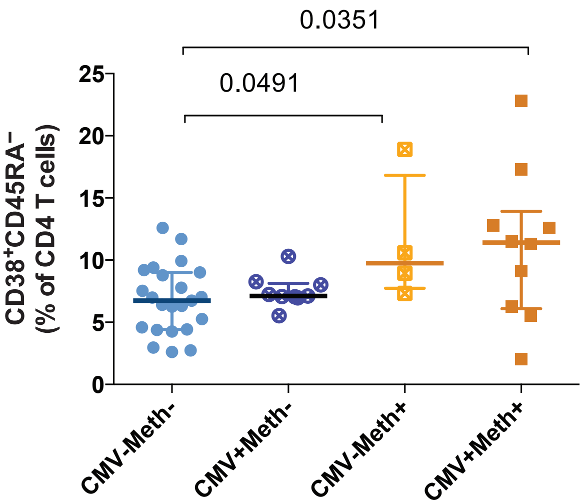

Supplementary Figure 2: Levels of CD4 T-cell activation (defined as CD38+CD45RA-) are shown in four groups stratified by Meth-usage and CMV shedding status. Individual and median values are shown. Two-sided p-values (Mann Whitney U test) are indicated.

Supplementary Table 1: Comparison of immune variables between recent meth user and control groups.

	Meth[1] n=11	Control[1] n=34	*P*-value[2]
Proliferation			
Ki-67$^+$ (% of CD4 T cells)	4.3 [3.3-5.0]	3.0 [2.4-4.2]	***0.032***
Ki-67$^+$ (% of Tn CD4 T cells)	2.2 [1.9-5.0]	1.4 [1.0-2.2]	***0.021***
Ki-67$^+$ (% of Tcm CD4 T cells)	4.6 [3.0-5.2]	3.4 [2.5-4.3]	*0.12*
Ki-67$^+$ (% of Ttm CD4 T cells)	4.5 [3.2-4.9]	3.7 [2.6-4.7]	*0.28*
Ki-67$^+$ (% of Tem CD4 T cells)	5.0 [3.8-7.9]	4.1 [2.9-5.5]	*0.23*
Ki-67$^+$ (% of CD8 T cells)	5.9 [3.7-7.1]	2.6 [2.2-3.6]	***0.0007***
Ki-67$^+$ (% of Tn CD8 T cells)	4.4 [2.0-7.0]	1.6 [1.0-2.7]	***0.0094***
Ki-67$^+$ (% of Tcm CD8 T cells)	7.5 [3.8-9.8]	3.1 [2.5-4.8]	***0.007***
Ki-67$^+$ (% of Ttm CD8 T cells)	4.9 [3.1-8.7]	2.6 [2.0-3.5]	***0.0054***
Ki-67$^+$ (% of Tem CD8 T cells)	5.4 [3.9-6.6]	2.6 [2.0-3.7]	***0.002***
Immune activation			
HLA-DR$^+$CD38$^+$ (% of CD4 T cells)	3.1 [2.2-5.9]	2.6 [1.9-3.3]	*0.28*
CD45RA$^-$CD38$^+$ (% of CD4 T cells)	11.0 [6.9-17.7]	7.0 [5.3-8.7]	***0.02***
HLA-DR$^+$CD45RA$^-$ (% of CD4 T cells)	8.5 [5.1-19/4]	7.1 [6.1-10.7]	*0.28*
HLA-DR$^+$CD38$^+$ (% of CD8 T cells)	12.8 [8.0-21.9]	18.7 [10.0-24.0]	*0.45*
CD45RA$^-$CD38$^+$ (% of CD8 T cells)	11.4 [7.5-20.0]	13.2 [5.7-16.6]	*0.73*
HLA-DR$^+$CD45RA$^-$ (% of CD8 T cells)	18.5 [10.6-28.6]	25.4 [16.7-32.0]	*0.16*
Exhaustion			
PD-1$^+$ (% of CD4 T-cells)	0.6 [0.04-2.9]	0.06 [0.02-0.14]	***0.02***
PD-1$^+$ (% of CD8 T-cells)	0.9 [0.5-1.2]	1.0 [0.5-1.8]	*0.7*
HIV reservoir			
LogPol (copies/10^6 cells)	2.1 [1.8-2.6]	1.9 [1.1-2.2]	*0.18*

[1]Median [IQR] are shown. [2]Mann Whitney U test between meth and non-meth users
